# Patrolling human SLE haematopoietic progenitors demonstrate enhanced extramedullary colonisation; implications for peripheral tissue injury

**DOI:** 10.1038/s41598-021-95224-y

**Published:** 2021-08-03

**Authors:** Ioannis Kokkinopoulos, Aggelos Banos, Maria Grigoriou, Anastasia Filia, Theodora Manolakou, Themis Alissafi, Nikolaos Malissovas, Ioannis Mitroulis, Panayotis Verginis, Dimitrios T. Boumpas

**Affiliations:** 1 Laboratory of Autoimmunity and Inflammation, Biomedical Research Foundation of the Academy of Athens, Athens, Greece; 2 Developmental Biology, Biomedical Research Foundation of the Academy of Athens, Athens, Greece; 3 The 4th Department of Medicine, Attikon University Hospital, National and Kapodistrian University of Athens Medical School, Athens, Greece; 4 Rheumatology-Clinical Immunology Unit, Medical School, University of Cyprus, Nicosia, Cyprus; 5 First Department of Internal Medicine, University Hospital of Alexandroupolis, Alexandroupolis, Greece; 6 Medical School, University of Crete, Heraklion, Greece; 7 Immunobiology and Developmental Biology Laboratories, Centre for Translational Research, Biomedical Research Foundation of the Academy of Athens (BRFAA), Soranou Efesiou 4, 11527 Athens, Greece

**Keywords:** Systemic lupus erythematosus, Haematopoietic stem cells

## Abstract

Systemic lupus erythematosus (SLE) is an autoimmune disease where bone-marrow-derived haematopoietic cells have a key role in its pathogenesis with accumulating evidence suggesting an aberrant function of haematopoietic stem/progenitor cells (HSPCs). We examined whether patrolling HSPCs differ from bone-marrow HSPCs both in SLE and healthy individuals, and how they participate in peripheral tissue injury. By employing next-generation RNA sequencing, the transcriptomes of CD34^+^ HSPCs deriving from the bone marrow and those patrolling the bloodstream of both healthy and individuals with SLE were compared. Patrolling SLE and Healthy human HSPC kinetics were examined through their inoculation into humanised mice. Patrolling and bone-marrow HSPCs have distinct molecular signatures, while patrolling SLE HSPCs showed an enhanced extramedullary gene expression profile. Non-mobilised, SLE-derived circulating HSPCs demonstrated altered homing capacities. Xenotransplantation of circulating HSPCs in humanised mice showed that human peripheral blood HSPCs possess the ability for extramedullary organ colonisation to the kidneys. Circulating and bone marrow-derived HSPCs are distinct in steady and diseased states. Patrolling SLE CD34^+^ HSPCs are able to home at extramedullary sites such as the spleen and kidneys, potentially participating in peripheral tissue injury.

## Introduction

Long-term hematopoietic stem cells (LT-HSC) differentiate through short-term HSC (ST-HSC) and then multipotent haematopoietic progenitor (MPP) stages into lineage-restricted progenitors such as lymphoid, myeloid, or megakaryocyte/erythroid progenitors. HSCs are an integral part of the immune response with the ability to sense inflammatory stimuli in infectious and chronic inflammatory diseases. Importantly, prolonged exposure to inflammatory stimuli during chronic inflammatory diseases has long-lasting effects on the Bone Marrow (BM) cell output’s nature through epigenetic modifications in hematopoietic stem and progenitor cells (HSPCs)^1–5^. Dysregulation of HSPC activity in the BM has been reported in several chronic inflammatory diseases, including inflammatory bowel disease, experimental spondyloarthritis, atherosclerosis and systemic lupus erythematosus (SLE)^6–9^.

SLE is the prototypic systemic autoimmune disease characterized by inflammation and damage in several organs and a disease course, where periods of treatment-induced remissions alternate with flares. In SLE, most cells participating in the pathogenesis of SLE originate from BM HSPCs. Murine and human SLE HSPC’s gene expression program is biased towards myelopoiesis and GMP progenitors^10,11^. Based on these findings we reasoned that in lupus, systemic inflammation may enhance both medullary and extramedullary myelopoiesis to meet the increased demand for effector cells in the periphery. In this setting, HSCPs may emigrate from the bone marrow to peripheral tissues and seed the red pulp of the spleen or other inflamed tissues to produce myeloid cells (neutrophils and monocytes) and contribute to peripheral pathology^11^.

Herein, we investigate how HSC in the BM and patrolling HSPCs in the circulation respond to lupus systemic inflammatory environment^12^. To this end, we collected BM and PB samples from healthy subjects and SLE patients and performed mRNA-seq in isolated human CD34^+^ HSPCs. Using HSC human surface markers, we confirm the presence of HSC in the PB, with the fast-cycling MPPs being increased in the PB of SLE individuals. Our analysis also indicates that PB- and BM-derived CD34^+^ progenitors have distinct gene expression signatures in transcriptional networks and cellular functions both in healthy and SLE patients, as well as different migratory and metabolic attitudes, between them. We also identify that the histone deacetylase *SIRT7* that has recently been shown to be relevant to HSC rejuvenation and reconstitution^13–15^, is highly expressed in PB-HSCs in comparison to their BM-counterparts. Using adult humanised mice as hosts, we report that human PB SLE CD34^+^ and MPP cells show altered homing and enhanced extramedullary colonisation in humanised mice compared to Healthy PB counterparts. SLE CD34^+^ HSPCs homing at extramedullary sites such as the spleen and kidneys may participate in local pathology.

## Results

SLE involves the dysregulation of the HSCs, resulting in a multi-organ disease phenotype^10^ that could potentially involve circulating patrolling progenitors. To this end, we isolated total RNA from magnetically-isolated CD34^+^ cells extracted from the periphery (PBMCs) and the bone marrow aspirates [bone marrow mononuclear cells (BMMCs)] of age-matched healthy and SLE individuals and subjected them to NGS mRNA-seq. All DEG from each comparison were used as input on downstream analyses, using multiple pathway databases for maximum enrichment and predictive modelling^16^.

### Human PB CD34^+^ HSPCs differ from BM CD34^+^ HSPCs

**Figure 1:**
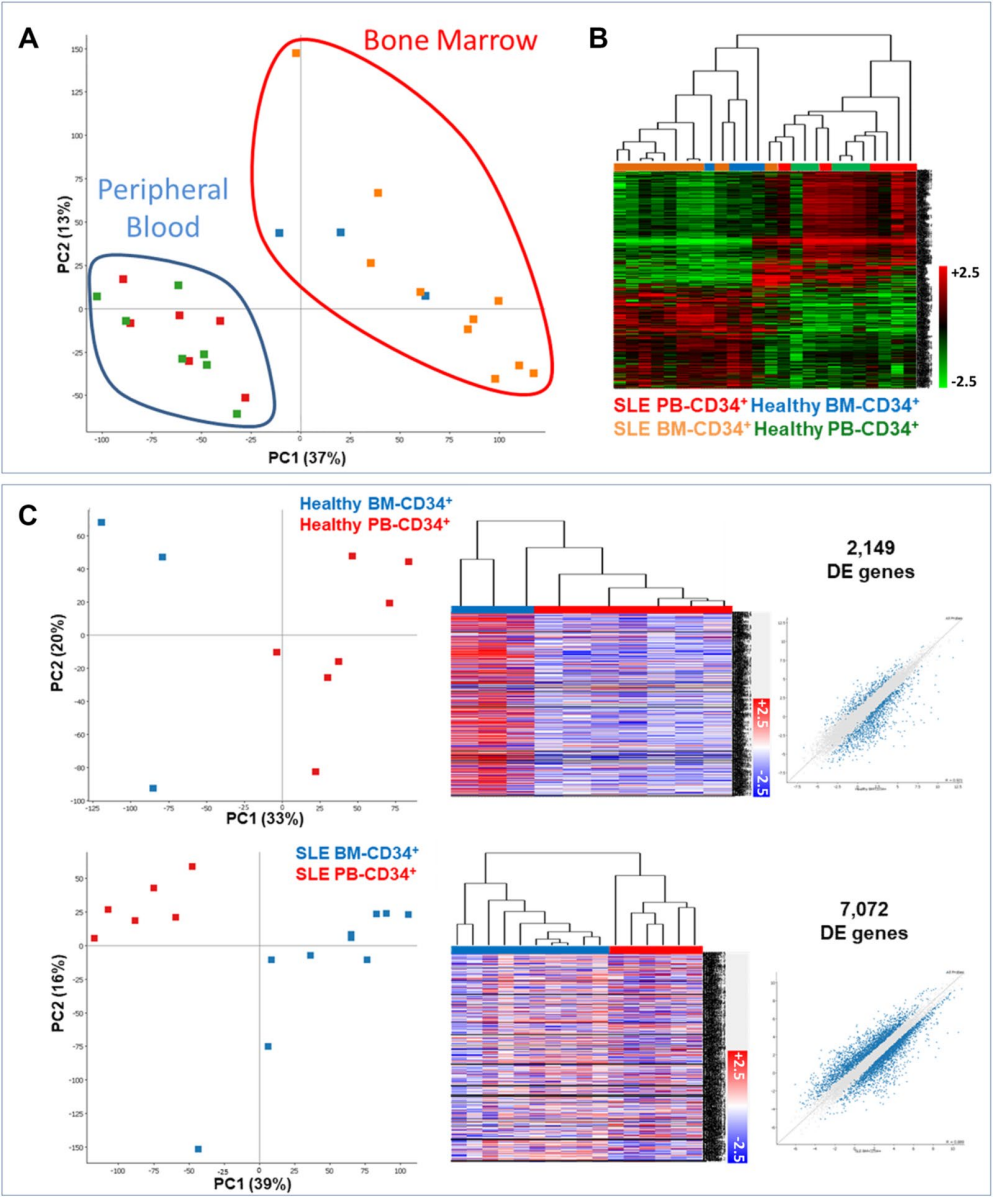
Circulating and niche haematopoietic progenitors have different transcriptomic signatures, irrespective of disease status. (**A**) PCA analysis indicating the transcriptional clustering of human CD34^+^ progenitors from SLE PB (red squares, n = 6), SLE BM (orange squares, n = 10), Healthy BM (blue squares, n = 3) and Healthy PB (green squares, n = 7). (**B**) Heatmap representation of 12,659 DEG depicting clustering of BM- and PB-derived haematopoietic progenitors in Healthy and SLE individuals. (**C**) PCA analysis, heatmap and scatter plots indicating the transcriptional clustering of human CD34^+^ progenitors from Healthy PB versus BM and SLE PB versus BM. EdgeR analysis indicated 2149 DEG between BM and PB CD34^+^ progenitors, while in SLE samples, 7072 DE genes were detected between PB and BM (FDR < 0.05).

We initially assessed whether the molecular signatures are distinct among the four haematopoietic progenitor populations (PB-SLE, PB-Healthy, BM-SLE and BM-Healthy). For this, we performed RNA-seq analysis in a human CD34^+^ cells from 26 human samples in total (3 healthy BM, 7 healthy PB, 6 SLE PB and 10 SLE BM). Principal component analysis (PCA) depicting all the DEG between those four groups, indicated distinct patterns in the transcriptome between BM- and PB-derived CD34^+^ progenitors, irrespective of disease status (four groups, Fig. 1A,B). This finding was also recapitulated when PB- and BM-derived progenitors were compared within the same disease setting (healthy or SLE, Fig. 1C, Supplementary Tables 1 and 2).

Next, we set to explore if transcriptome analysis could reveal significant gene expression differences in CD34^+^ HSPCs, due to disease status (i.e. Healthy vs SLE). PCA analysis showed that PB comparison (in contrast to BM comparison) did not show strong segregation, bearing a smaller number of DEG (Supplementary Fig. 1A,B).

**Figure 2:**
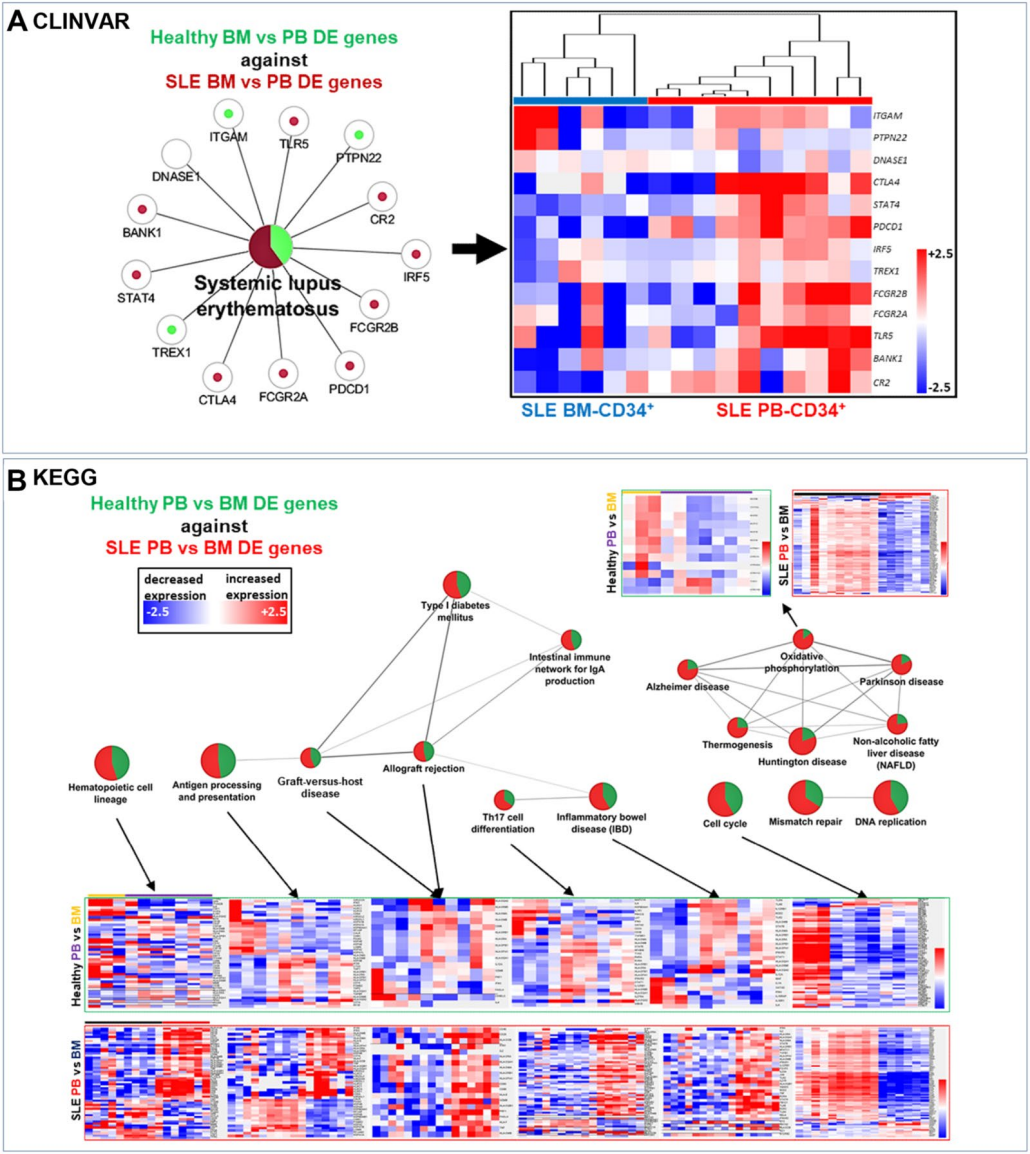
Different transcriptomic signatures in SLE PB and BM progenitors. (**A**) DEG between SLE BM-derived and PB-derived progenitors were used as an input on CLINVAR database, using as a reference cut-off the DEG found between healthy BM-derived and PB-derived progenitors. Graphical representation showing genes found to be either enriched in SLE (dark red) or Healthy (light green) progenitors, from genes reported in the CLINVAR database (*p* = 0.049). A heatmap of these DE genes indeed indicated higher expression levels in SLE PB-derived CD34^+^, in comparison to SLE BM-derived CD34^+^. (**B**) KEGG pie chart analysis of DEG between SLE-derived BM and PB CD34^+^ progenitors (red), and Healthy BM vs PB-derived (green). Pie chart connecting lines indicate kappa-score relations. Heatmaps indicating DEG that shown to be enriched in Healthy-derived PB (purple) when compared to Healthy-derived BM (light orange) progenitors, and SLE-derived PB (red) when compared to SLE-derived BM (black) progenitors. All analyses were performed with an FDR < 0.05 (Bonferroni and Heidelberg) correction.

We looked further into this comparison by comparing the gene expression analysis of SLE DEG between PB and BM, by using the Healthy DEG PB versus BM output as a reference point (homeostatic cut-off). CLINVAR analysis provided a unique SLE-specific signature, providing a list of genes (and potentially their variants) altered in PB SLE CD34^+^ HSPCs (Fig. 2A). Hierarchical clustering revealed that these genes were indeed differentially expressed in PB SLE versus PB Healthy progenitors. KEGG pathways analysis revealed that antigen presentation and processing that included Graft-versus-Host-Disease (GvHD), allograft rejection and oxidative phosphorylation, were overrepresented in SLE compared to healthy counterparts (Fig. 2B). Haematopoietic cell lineage, Th_17_ cell differentiation, antigen processing and presentation as well as cell cycle, were also enriched in the PB setting, with the majority of genes being elevated (except cell cycle GO) in the SLE PB-derived cell populations, in comparison to healthy PB-derived progenitors. These data pinpoint a potential discrepancy between SLE PB- and BM-derived CD34^+^ cells that may cause significant differences in progenitor mobilisation and immune activation^17,18^. KEGG analysis of DE genes in SLE versus healthy CD34^+^ cells showed that genes involved in allograft rejection pathways were enriched in the SLE setting when compared to healthy CD34^+^ HSPCs (Fig. 2B).

Together, these analyses underscore the transcriptome differences in the circulating HSPCs in relation to BM CD34^+^ progenitors while underpinning their potential implication in SLE disease pathogenesis.

### Human SLE PB HSPCs show an enhanced migration gene expression profile

The CLINVAR database for genes involved in human disease^19,20^ indicated that SLE haematopoietic circulating progenitors differentially expressed key genes being involved in pro-inflammatory and immune-regulatory functions, including cell mobilisation [*IFN-γ, IL-8R (CXCR1), CCL3L1-CCL3*], as well as in SLE-specific immune regulation (*CTLA4, STAT4, PCD1*), when compared to Healthy PB circulating progenitors (Supplementary Fig. 2A). REACTOME Reactions and Pathways analyses^21^ revealed significant changes in gene expression involved in extracellular matrix (ECM) organisation, immune system activation through CD3, and cytokine/chemokine regulation that involved IL-10 signalling and trafficking in inflammation sites^12,22^, with the majority of genes in the SLE-derived PB progenitors, being upregulated in comparison to Healthy-derived PB (Supplementary Fig. 2B). Finally, KEGG pathways analysis^23^ was indicative of DEGs that involved the migratory and extramedullary increased potential (cytokine and cytokine-receptor interactions) as well as altered immune activation of circulating SLE progenitors when compared to their circulating Healthy progenitor counterparts (Supplementary Fig. 2C).

By employing the same analytical principle (CLINVAR, REACTOME Reactions/Pathways and KEGG) on DEG between Healthy versus SLE BM CD34^+^ progenitors, although a similar immune reaction profile was evident, an extramedullary/migratory signature was not readily apparent in the SLE BM CD34^+^ HSPCs, as judged by cytokine and cytokine-receptor interaction gene expression profile (Supplementary Fig. 3A–C). CLIVAR and KEGG analyses on DEG between Healthy PB versus BM and SLE PB versus BM CD34^+^ progenitors indicated that SLE circulating progenitors showed a decrease in oxidative phosphorylation gene expression (Supplementary Fig. 4A). Healthy BM progenitors had a non-migratory profile when compared to Healthy PB progenitors (Supplementary Fig. 4B).

Together, these results indicate that human lupus circulating HSPCs possess a transcriptomic signature that may promote an altered migration potential in relation to BM HSPCs.

### Specific transcription factor binding sites predicted in PB- and BM-derived CD34^+^ progenitors

**Figure 3:**
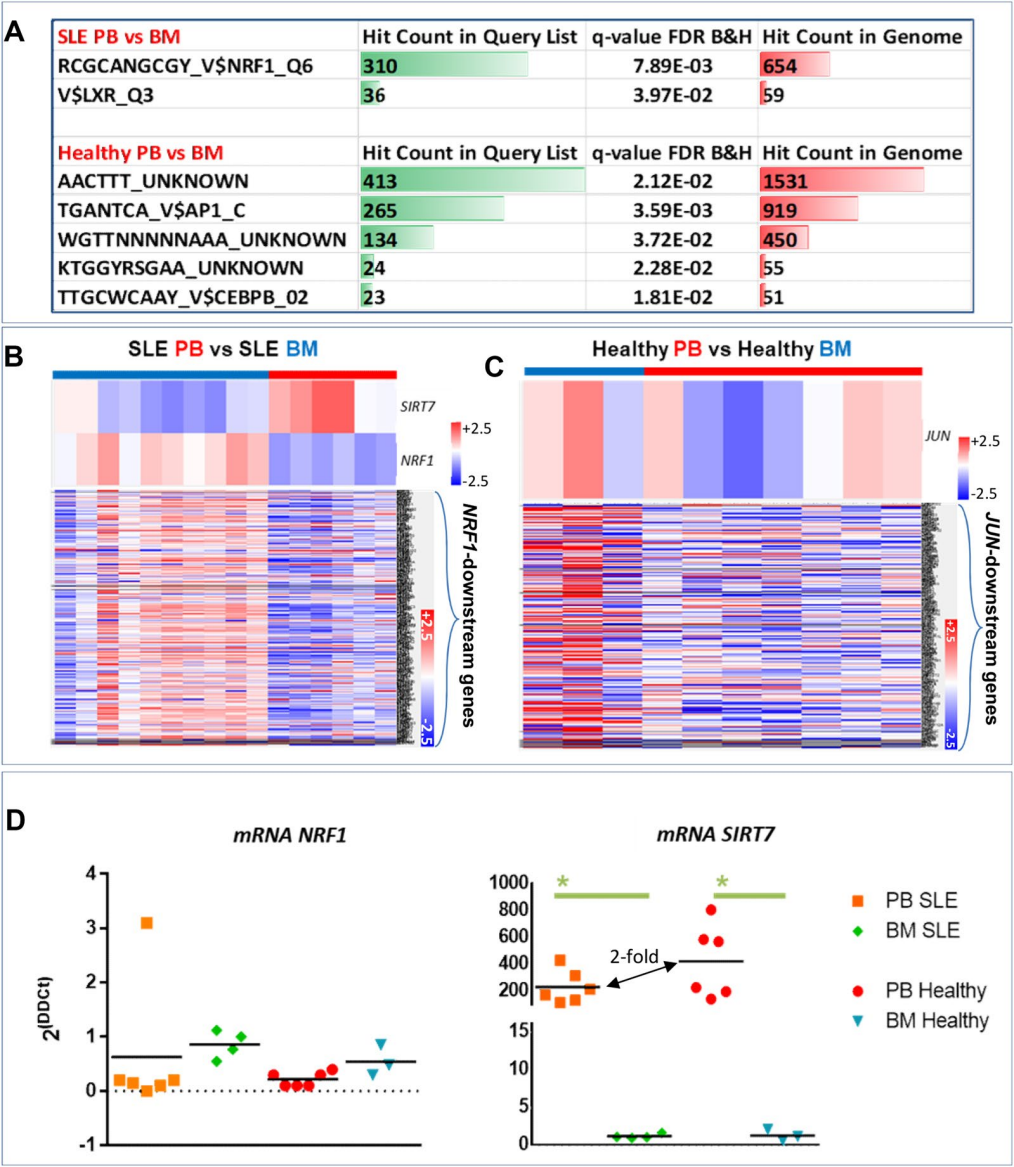
The *NRF1-SIRT7* transcriptional signature is altered in PB CD34^+^ progenitors. (**A**) A table indicating the predicted TF binding sites (Toppgene™ software) based on the DEG input list. In the SLE PB versus BM comparison the *NRF1* and *LXR* TFs were identified, while in the Healthy PB versus BM comparison, *AP1* (*JUN*) and *CEBPB* were identified along those with unknown TF bindings site motifs. (**B**) Heatmaps of *SIRT7* and *NRF1* expression in SLE PB and SLE BM, along with 310 DEG that are affected by *NRF1* expression. (**C**) Heatmaps of *JUN* expression in Healthy PB and Healthy BM, along with 265 DEG that are affected by *JUN* expression. (**D**) Real-time PCR graphs for *NRF1* (ENST00000353868.5, ENST00000393230.6 isoforms) and *SIRT7* (ENST00000575360.5, SIRT7-209, non-sense mediated decay) in all four groups. The expression of *NRF1* was not statistically different in any of the four groups, albeit there was a trend of lower expression in the SLE PB. In contrast, *SIRT7* expression was vastly and significantly elevated in PB CD34^+^ progenitors, in comparison to BM CD34^+^ progenitors. One-way ANOVA with Bonferroni’s posthoc test, *p < 0.05, n = 3–6.

Next, we sought to identify potential Transcription Factor (TF) binding sites based on the DE genes identified between PB and BM, in both healthy and SLE patient samples. The analysis revealed a non-exhaustive list of potential TF sites which pinpointed unique TFs that may regulate a cohort of gene regulation (Fig. 3). SLE PB-derived progenitor DEGs revealed a marked upregulation in *SIRT7* and a downregulation in *NRF1 and NRF1-dependent* DEGs (131 genes), while *LXR (NR1H3)*-dependent gene expression showed mixed DEG patterns (36 genes), when compared to SLE BM-derived HSPCs (Fig. 3A,B and Supplementary Fig. 5A).

Due to the dynamic synergistic effect that *NRF1* induces on SIRT7 in relation to HSPCs^24,25^, isoforms of both *NRF1* and *SIRT7* were explored (Fig. 3D and Supplementary Fig. 5E). *SIRT7* expression of its nonsense-mediated decay isoform showed higher expression in PB, in relation to BM (both SLE and Healthy), yet with a two-fold decrease (non-statistically significant) in SLE PB, in comparison to Healthy PB. In healthy PB-derived progenitors, predicted expression of altered TFs *AP1 (JUN)—*and *CEBPB*-dependent DE genes produced mixed differential gene expression patterns, (265 and 23 genes, respectively, Fig. 3C and Supplementary Fig. 5B).

We then investigated the prospect of TFs that may be related to the DE genes on PB- and BM-derived HSPCs between SLE and healthy patients. Prediction analysis showed a cascade of TF-dependent DEGs in the BM CD34^+^ progenitors, but none in the PB CD34^+^ progenitors (Supplementary Fig. 5C,D). In most of the predicted TFs, DEGs were downregulated in the SLE BM-derived progenitors compared to controls (Supplementary Fig. 6). Yet, the TF family *E2F* and specifically *E2F1* (39 genes) revealed a cluster of DEGs that were upregulated in the SLE in comparison to healthy controls.

### SLE PB CD34^+^ progenitors showed an altered extramedullary differentiation in humanised mice

**Figure 4:**
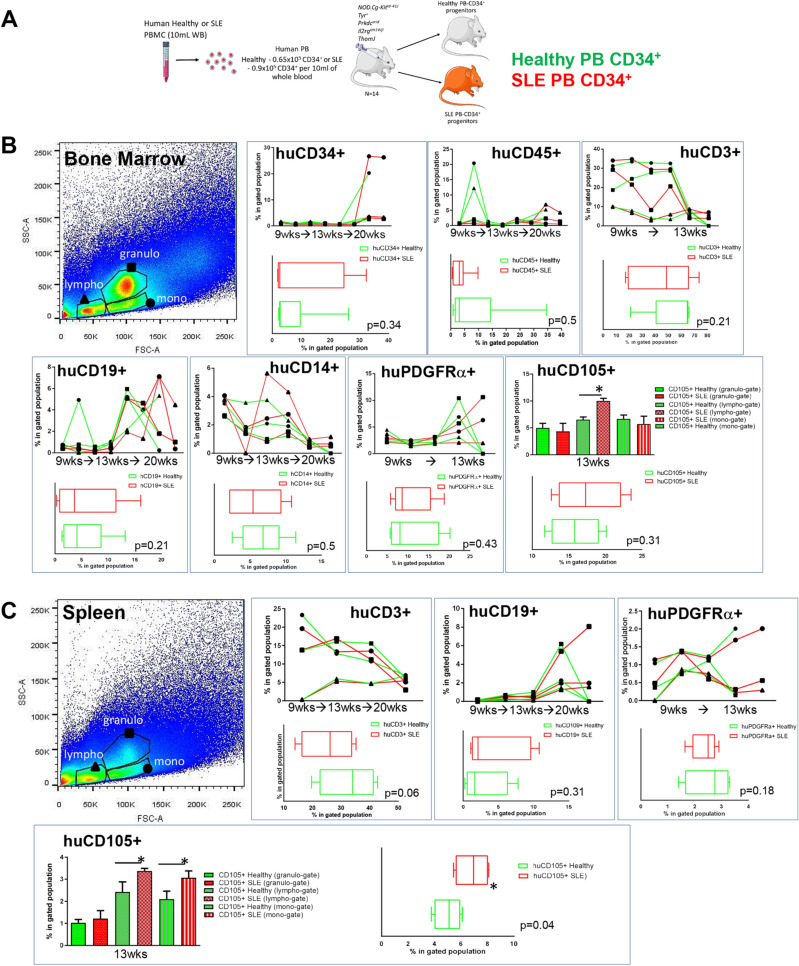
Altered kinetics of SLE CD34^+^ progenitors in BM and spleen. (**A**) Schematic representation of the xenotransplantation procedure of human PB CD34^+^ in 2–3-month-old mice, N = 14. (**B**) Mice were sacrificed at designated time intervals and the BM-derived cells were subjected into immunostaining with antibodies against humanonly surface markers, in order to assess human cell presence. (**C**) Mice were sacrificed at designated time intervals and the spleen-derived cells were subjected into immunostaining with antibodies against human-only surface markers, in order to assess human cell presence. Lympho-gates (triangle), granulo-gates (square) and mono-gates (circle) represent different immune cell subpopulations and therefore examined separately. Box plots indicate the average percentages of total lymphocyte populations on all time-points. Paired Student’s T-test.

**Figure 5:**
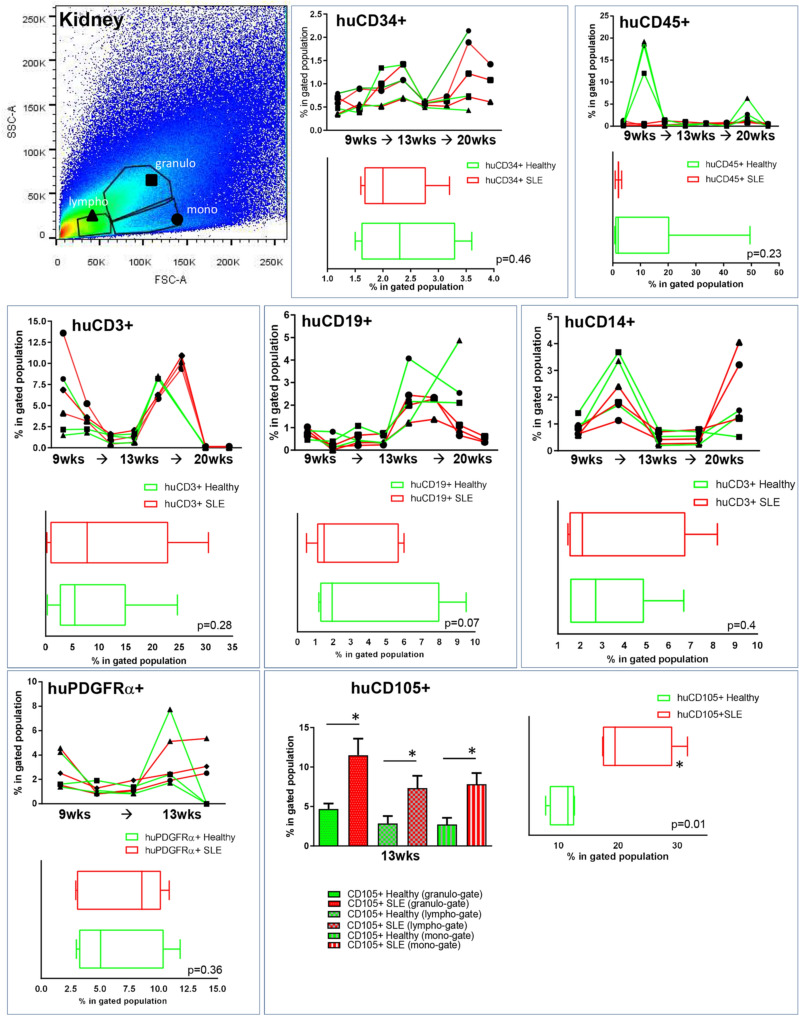
Altered kinetics of SLE CD34^+^ progenitors in the kidney. Mice were sacrificed at designated time intervals and the kidney-derived cells were subjected into immunostaining with antibodies against human-only surface markers, in order to assess human cell presence. Lympho-gates (triangle), granulo-gates (square) and mono-gates (circle) represent different immune cell subpopulations and therefore examined separately, according to their FSC/SSC readout. Box plots indicate the average percentages of total lymphocyte populations on all time-points. N = 14. Paired Student’s T-test.

Based on the potentially altered migratory transcriptional profile of circulating HSPCs, we interrogated the behaviour of human PB CD34^+^ progenitors as xenotransplants. Human SLE or Healthy 2.5 × 10^5^ CD34^+^ progenitors were injected into each of fourteen 2–3 month-old NBSGW mice (one human sample per mouse, 7 inoculated with SLE-derived and 7 with Healthy-derived PB CD34^+^ HSPCs, Figs. 4 and 5A). Mice were sacrificed at 9, 13, and 20-weeks post-injection to assess human cell colonisation potential. Mice did not develop alopecia, a characteristic of GvHD, up to 20-week post-injection^26^. We observed altered migration kinetics and lymphocyte/myeloid differentiation potential from SLE PB CD34^+^ human cells when compared to healthy PB CD34^+^ cells, in the murine BM (Fig. 4B).

In the spleen and kidneys differences in human CD19^+^, CD14^+^ and CD3^+^ cell percentages, between SLE- and Healthy-derived progenitors were evident (Figs. 4C and 5A). Of interest, a substantial number of human CD34^+^ was evident in the murine kidneys, indicating possible homing of secondary organs. We also examined whether HSPCs could have potentially adopted a BM niche-like profile^27–29^. We found that human SLE-derived PDGFRα^+^ and CD105^+^ cells were present and in an increased percentage in the murine BM, kidneys and spleen, in comparison to healthy-derived human PB progenitors (Figs. 4B,C and 5).

These xenotransplantation experiments indicate that human PB HSPCs mediated extramedullary colonisation with an increased ability of the SLE-derived HSPCs to home at extramedullary sites such as the kidneys, where they may participate in local pathology through the formation of primitive HSPC colonies (Fig. 5)^29^.

### Increased frequency of MPPs in the peripheral blood of SLE patients, with increased extramedullary colonisation in humanised mice

**Figure 6:**
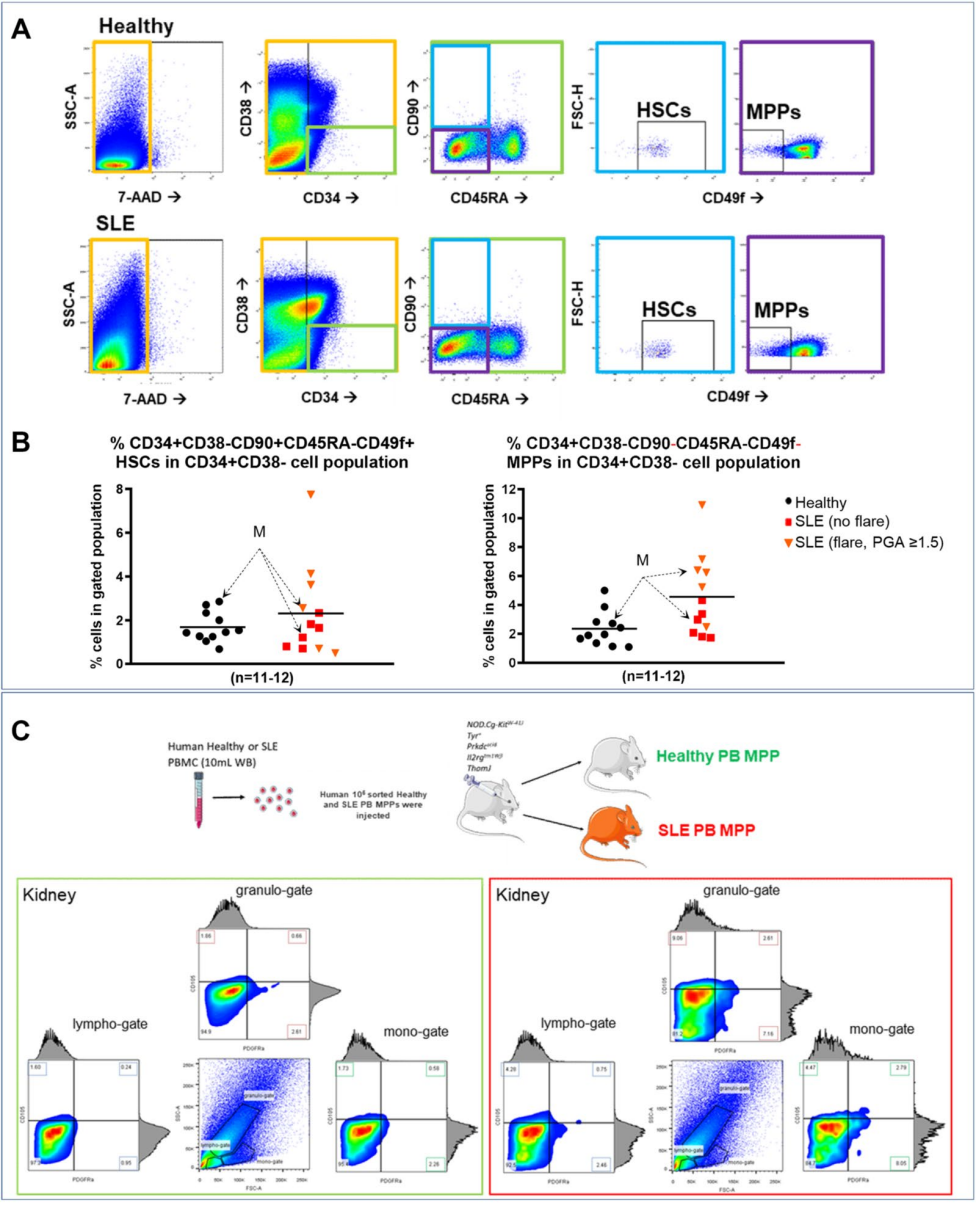
Peripheral blood MPP number is elevated in SLE and migrate preferentially to the kidneys. (**A**) Gating strategy for obtaining human HSC and MPP cell populations from PBMC from healthy (n = 11) and SLE (n = 12) individuals. These representative graphs from a total of gated live cell population (7AAD, orange gate) was initially gated for CD34^+^CD38- progenitor pool (green gate), which was further examined for CD45RA^−^CD90^+^CD49f^+^ HSCs (light blue gate) and CD45RA^−^CD90^−^CD49f^−^ MPPs (purple gate). (**B**) X–Y graphs showing the percentage of HSCs and MPPs in PBMC-gated and CD34^+^CD38^−^-gated cell populations (and mean values). There are statistically more MPPs in the CD34^+^CD38^−^-gated cell populations that is linked to SLE flare and PGA > 1.5, at the time of sample collection (orange triangles, *p* < 0.03, Student’s T-test). No statistical difference in HSC percentage between SLE and Healthy PB (*p* = 0.35, Student’s T-test). M = male subjects. (**C**) Mice were sacrificed at 5 weeks and kidney-derived cells were subjected into immunostaining with antibodies against human-only surface markers for CD105 and PDGFRα, in order to assess human cell presence. Lympho-gates, granulo-gates and mono-gates represent different immune cell subpopulations and therefore examined separately, according to their FSC/SSC readout N = 4.

**Figure 7:**
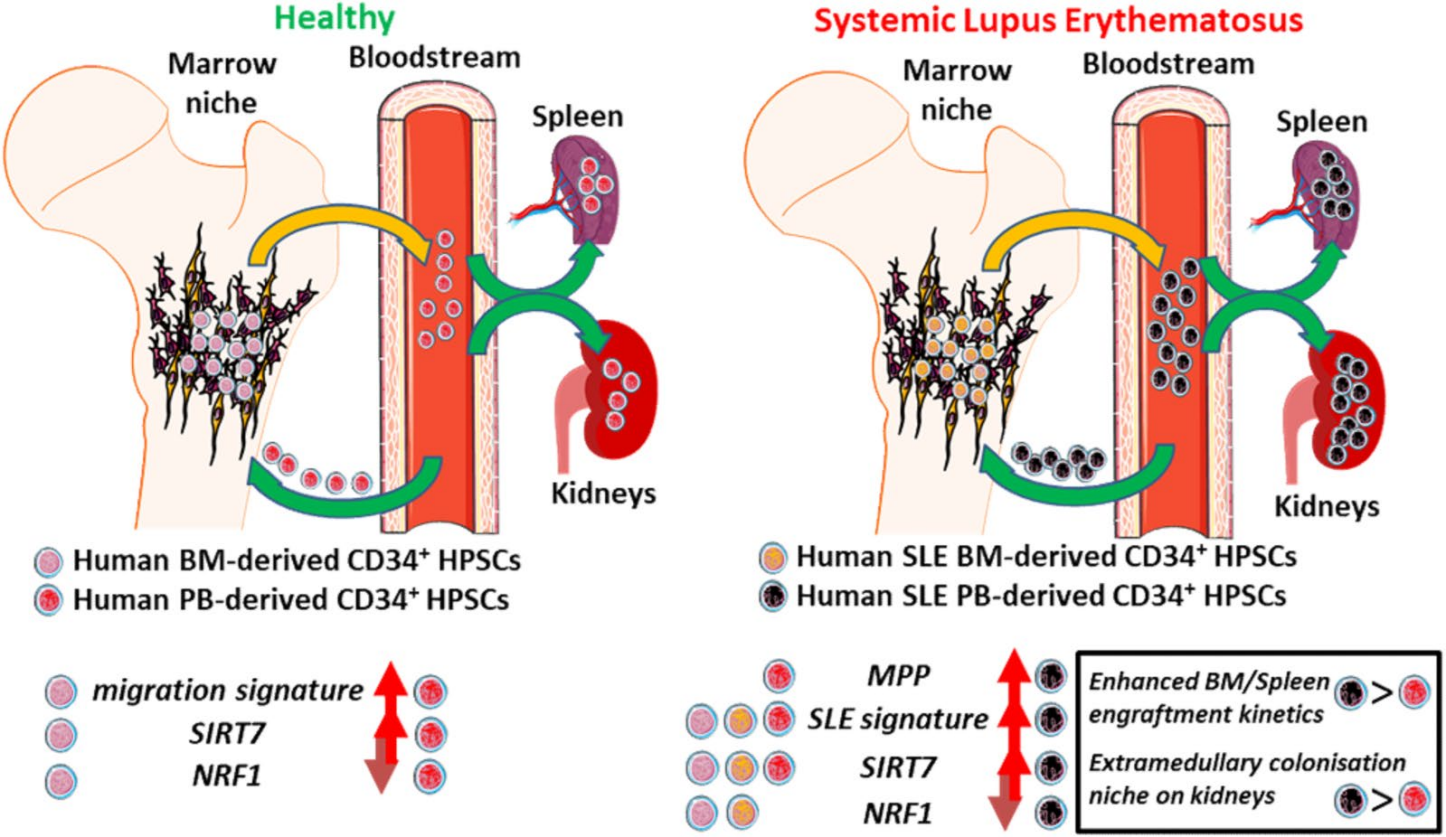
Schematic representation of the kinetics of PB- and BM-derived human CD34^+^ progenitors from healthy and SLE individuals, in relation to *NRF1* and *SIRT7* DEG as well as their extramedullary potential based on RNA-seq and comparison of SLE and Healthy PB xenotransplantation potential in humanised mice.

In order to assess whether HSCs/MPPs numbers (found within the CD34^+^ cell population) differ in the periphery of SLE patients, PBMCs were subjected to immunostaining against the surface markers CD34, CD38, CD45RA, CD90 and CD49f., along with the cell viability dye 7AAD. Parallel gating indicated the presence of well-defined HSCs and MPPs in PBMC derived from both SLE patients and Healthy individuals (Fig. 6A). On gated CD34^+^CD38^−^ populations, SLE MPPs were significantly more in BM and statistically significant more in PB (over a two-fold increase) when compared to healthy controls [Fig. 6B, and as we have shown recently concerning human healthy and SLE BM CD34^+^ transcriptomes^11^]. No statistical difference was observed in CD34^+^CD38^+^ cell populations (Supplementary Fig. 7A). In addition, SLE flares and a PGA ≥ 1.5, showed to be linked to a two-fold MPP increase in SLE PB (Fig. 7B and Table 1).

**Table 1:** Demographic and clinical characteristics of human SLE subjects at the time peripheral blood was obtained.

Age	Sex	PGA	SLEDAI	Flare	Main disease features	Previous medication
27	Female	2.5	6	Yes	Ulcer/arthritis/CNS/ANA+	ΑΖΑ
25	Female	3	18	Yes	Rash/arthritis/renal/CNS/ANA+	None
65	Female	3	12	No	Arthritis/ANA+/pericarditis	HCQ, AZA
37	Female	1.5	10	Yes	Rash/arthritis/CNS/ulcers/ANA+/alopecia/Hashimoto/peptic ulcer/asthma	None
50	Male	2	0	No	Rash/arthritis/CNS/ANA+/psoriasis/cataract	None
38	Female	2.5	10	No	Rash/arthritis/CNS/ulcers/ANA+/myositis/thrombophilia/lung nodules	ΜΤΧ, RTX, IVIg, IV-MP
41	Female	0	6	No	Ulcers/arthritis/ANA+/alopecia	ΜΤΧ
51	Female	2	4	No	Rash/arthritis/CNS/ANA+/alopecia/peptic ulcer	ΜΤΧ, ARAVA
76	Male	3	3	Yes	Rash/uclers/CNS/ANA+/haemolytic anaemia/thrombocytopenia/hypertension	CYC, IV-MP
37	Female	1.5	7	Yes	rash/arthritis/CNS/ulcers/Hashimoto/peptic ulcer/past	GI cancer none
55	Female	1	6	Yes	Rash/arthritis/CNS/ulcers/ANA+/alopecia	MTX
43	Female	2	7	Yes	Rash/arthritis/CNS/ANA+/Anti-DNA/Lung disease	None
40	Female	1.5	6	Yes	Rash/arthritis/CNS/ANA+	ΜΤΧ
74	Female	2	14	No	Rash/arthritis/CNS/ANA+/anti-DNA/thyroiditis	None
60	Female	1	0	No	Rash/arthritis/CNS/ulcers/ANA+/renal/anti-DNA/APS	CYC, AZA, IV-MP
40	Female	1	4	No	Rash/arthritis/ulcers/CNS/ANA+/anti-DNA	MTX, DMSO, IV-MP
38	Female	2.5	10+	Yes	Arthritis/CNS/ulcers/ANA+/history of leukopenia/Hashimoto/lung nodules/thrombophilia	ΜΤΧ, RTX, IVIg, IV-MP
74	Female	1.5	8+	No	Rash/arthritis/CNS/ANA+/anti-DNA/thryroiditis	None
32	Female	1	8	No	Rash/ulcers/renal/CNS/arthritis/ANA+/thrombocytopenia/anti-DNA/thyroid disease	RTX
36	Female	3	16	No	Rash/ulcers/CNS/arthritis/ANA+/	AZA
38	Female	2	12	No	Rash/ulcers/renal/CNS/arthritis/ANA+/alopecia/history of leukopenia	CYC, MMF
52	Female	1	4	Yes	Rash/ulcers/renal/CNS/arthritis/ANA+/alopecia/anti-DNA/APS	CYC, AZA, IV-MP
41	Female	1	4	No	Rash/ulcers/CNS/arthritis/ANA+/alopecia/Hashimoto/	MTX
58	Female	1.5	2	No	Rash/CNS/arthritis/ANA+/history of leukopenia/anti-DNA/Hashimoto/	HCQ, MTX, CYC, T4, Efexor, Bactrimel
52	Female	2	4	Yes	Rash/CNS/arthritis/ANA+/peptic ulcer	MTX, ARAVA
48	Female	1	8	No	Rash/ulcers/CNS/arthritis/alopecia/ANA+/hamelytic anaemia/leukopenia/anti-DNA	HCQ, MTX, AZA
45	Female	1.5	6	No	Rash/CNS/arthritis/ANA+/APS/cardiovascular disease	None
64	Female	0.5	4	No	Ulcers/CNS/arthritis/renal/ANA+/APS/Hashimoto/Sjögren's/	CYC
62	Female	0.5	4	No	Ulcers/CNS/arthritis/ANA+	MTX, CELESTONE
53	Female	0.5	4	No	Rash/CNS/arthritis/ANA+/thrombocytopenia/	CYC, AZA, IV-MP
49	Female	1.5	6	No	Rash/ulcers/CNS/arthritis/renal/ANA+/Hashimoto	MTX/ARAVA
47	Female	0.5	4	Yes	Rash/arthritis/renal/CNS/ANA+/history of leukopenia/anaemia	None
65	Female	2	8	No	CNS/ANA+/history of leukopenia/thrombocytopenia/RA	CYC, AZA
73	Female	1.5	6	No	rash/CNS/arthritis/ANA+/alopecia/anti-DNA/cataract	CYC, AZA
61	Female	2	6+	No	CNS/arthritis/ANA+/alopecia/renal/PNS/diabetes/adrenal	HCQ, MTX, RTX
60	Female	1.5	6	Yes	Rash/CNS/arthritis/ANA+/alopecia/Hashimoto/diabetes	HCQ
52	Female	1.5	14	Yes	Rash/myelopathy/arthritis/CNS/ANA+/Hashimoto/cardiomyopathy	None
29	Male	1.5	10	Yes	Arthritis/rash/alopecia/history of leukopenia/fever/anaemia	None

*HCQ* hydroxychloroquine, *PZ* prednisone, *AZA* azathioprine, *MTX* methotrexate, *RTX* rituximab, *CYC* cyclobenzaprine, *IV-MP* intravenous methylprednisolone, *ARAVA* Leflunomide, *IVIg* intravenous immunoglobulin, *MMF* Mycophenolate mofetil, *DMSO* dexamethasone, *ANA* anti-nuclear antibodies, *CNS* central nervous system, *RA* rheumatoid arthritis, *PGA* physician global assessment.

In light of this, SLE (derived from patients that exhibited SLE flares with a PGA ≥ 1.5) or Healthy MPPs were also injected into each of four 2-month old NBSGW mice, and assessed for colonisation potential in the BM, spleen and kidneys, 5 weeks later (one human sample per mouse, Fig. 6C and Supplementary Fig. 7B). FACS analysis indicated that MPPs colonise neither the bone marrow nor the spleen (Supplementary Fig. 8). In contrast, only the kidneys were occupied by human MPP-derived CD105^+^ and PDGFRα^+^ cells, with SLE-derived cells showing an increased presence, when compared to Healthy-MPP-derived cells.

## Discussion

Dysregulation of HSPC activity in the BM has been reported in several chronic inflammatory diseases, including inflammatory bowel disease, atherosclerosis and in SLE. Here, we investigated the potential gene expression differences that involve altered migration between PB and BM CD34^+^ HSPCs from both Healthy and SLE patients.

We confirm that PB CD34^+^ HSPCs are distinct from BM CD34^+^ HSPCs as previously shown in a study relying on surface marker intensity alone^30^. We show that the transcriptional landscape is vastly different between BM and PB HSPCs, likely underlying altered outcomes concerning extramedullary colonisation and engraftment success^31^. The authors of the latter study also pinpointed, as we did, to the importance of *E2F* TF family gene expression in cell cycle progression. This could be why we see an increase in the number of MPPs in the SLE setting, as confirmed in an SLE murine model in our laboratory^11^ (and recent unpublished observations). In fact, what we observed was that PB HSPCs have decreased oxidative phosphorylation and cell cycle signatures as compared to BM HSPCS, indicating a more activated haematopoiesis along with a reduced self-renewal potential^32^.

In the SLE setting, PB HSPCs have a gene expression signature that could exacerbate inflammatory responses at local tissues such as the kidneys and other target organs in SLE such as joint and skin. This could be because a number of relevant transcriptional pathways is altered in PB HSPCs, in comparison to BM HSPCs. Of interest, the TLR-7/TNF-alpha/IFN-gamma axis that has been shown recently to be involved in extramedullary damage in murine SLE was shown to be highly upregulated in our human PB HSPCs, and especially in SLE PB HSPCs, when compared to BM HSPCs (data not shown)^33^. In humanised mice, xenotransplanted SLE-PB HSPC-derived cells were more prone to colonise extramedullary organs such as kidneys but not the BM nor spleen [possibly due to decreased oxidative phosphorylation and self-renewal capabilities^32^], when compared to Healthy-PB HSPCs. SLE-PB CD34^+^ and MPP progenitors may participate in local inflammatory reactions in the periphery due to their increased homing potential and altered T_h_ activation^34^ while could be promoting an aberrant haematopoietic “niche”^35,36^. Indeed, Noroozinia A. et al*.* recently demonstrated the presence of human interstitial CD34^+^ cells in patients diagnosed with the active phase of lupus nephritis^37^. Additionally, chimeric human CD3^+^CD34^+^ cell populations have been identified in human kidneys with active SLE, similar to the findings in our xenotransplantation experiments^38^. A future proof-of-concept experiment would be to fluorescently tag human SLE PB-derived HSPCs prior to inoculation and assess their presence in organs of interest through immunohistochemistry.

Of particular interest is *NRF1* TF, a master mitochondrial regulator^39^, which showed a marked decrease in SLE PB, in comparison to SLE BM (but not healthy PB, data not shown). NRF1 is implicated in pathways related to mitochondrial biogenesis and systemic chronic inflammation, including SLE^40,41^. A recent publication indicated that SIRT7, a nutrient-sensing protein and a histone deacetylase, when bound to NRF1, is able to alter essential HSC functions related to ageing, possibly via mitochondrial function^15^ and/or repopulation potential via invoking genome stability (when located to the nucleus)^13,42^. We did observe a multi-fold increase in *SIRT7* gene expression, in PB-derived cells, in comparison to BM-derived cells, and a global decrease in oxidative phosphorylation gene expression (Figs. 4B and 5D, respectively). These events could be linked to a reduced HSC quiescence with altered regenerative and bone-marrow reconstitution capabilities^13,14,24,25^ and, as far as SLE is concerned, direct multi-organ autoimmune inflammation^43^ through extramedullary colonisation. Our results corroborate those of a recent study where *E2F1* was identified as one of the susceptibility loci in SLE, directly involving *NRF1* in Asian and European human populations (data not shown)^40^. Of note, *SIRT7* and *NRF1* genes were inversely, but not differentially, expressed in Healthy PB versus BM CD34^+^ HSPCs.

A recent study pinpointed that *SIRT1* is involved in recurring infections in SLE patients^44,45^; SIRT7 has the ability to bind directly to SIRT1, possibly being involved in lupus nephritis^46^. Recently, Alexander Kaiser et al*.* reported that SIRT7 levels influence remission responses after HSPC transplantation in myeloid leukaemia^47^. As such, the aforementioned SIRT could be one of the potential epigenetic targets for alleviating peripheral tissue injury in SLE^48^. Indeed, ongoing experiments will focus on investigating if this protein complex confers substantial changes to HSC’s reconstitution capability and other properties such as multi-organ extramedullary colonisation conferring organ-specific SLE tissue injury (model proposed for this study, Fig. 7). To our knowledge, this is the first study that explores the transcriptome of human non-mobilised circulating HSPCs in the context of a systemic autoimmune disease.

We recognise that an important limitation of the current study is that the expression profile of bulk CD34^+^ HSPCs cannot disentangle minor shifts in the transcriptome of progenitor subpopulations. We have shown that from all the major HSPC subpopulations examined primarily with CD34 and CD38 surface markers, MPPs, but not HSCs, were significantly enriched in SLE-derived PBMCs, when compared to Healthy-derived PBMC. Investigating more human HSPC subpopulations using a larger combination of cell surface markers should potentially reveal novel subgroups that persist in the SLE setting. Yet, this study does pave the way for revealing clinically important transcriptomic differences and migration kinetics between niche and patrolling HSPCs in humans; these findings need to be investigated further using single-cell mRNA-seq and/or mass cytometry technologies.

In summary, we show here that the human PB CD34^+^ HSPC transcriptome differs substantially from that of human BM CD34^+^ HSPC, leading to potentially altered homing capabilities. These findings underscore the need for studying further non-mobilised PB CD34^+^ cells for haematopoietic stem cell therapy (HSCT) regimes, since they may be more beneficial in some autoimmune disease treatments, including SLE, as reported recently^34^. These data also shed light to a pathway that involves TF *NRF1* and histone deacetylase *SIRT7* in SLE. Finally, we also demonstrate for the first time that in an autoimmune/inflammatory disease, human HSPCs are not only activated and circulating, but they are also able to migrate and survive in places other than the BM such as the spleen, a site of peripheral immune responses in SLE, as well as at sites of tissue damage, such as kidneys.

## Methods

### Animals

The NBSGW humanised mouse strain was purchased from Jackson Laboratories (JAX Stock No. 026622) and maintained as homozygotes (*NOD.Cg-Kit*^*W-41J*^*Tyr*^+^*Prkdc*^*scid*^*Il2rg*^*tm1Wjl*^*/ThomJ*). All animals used were 2–3 months of age upon the time of xenotransplantation experiments and fed chow diet in germ-free housing conditions. All mouse animal work and experimental protocols have been approved by the BRFAA ethics committee and the Attica Veterinary Department (758634/22-11/2019). The study is reported in accordance with ARRIVE guidelines.

### Isolation of human CD34^+^ progenitors from BM and PB

Human BM aspirates and PB (10 ml each) were collected in EDTA-coated tubes from healthy and SLE patients and subjected to density gradient centrifugation using Histopaque-1077 (Sigma-Aldrich). Briefly, blood was diluted 1:2 with PBS (for PB) and 1:3 PBS (for BM) and carefully layered over Histopaque medium. Tubes were centrifuged at 400 g for 30 min (no break) at room temperature. White blood cell layer was carefully collected, and cells were washed with PBS, and treated with red-blood cell lysis buffer (Biolegend, cat no. 420301) prior to magnetic bead separation in order to obtain a minimum of 90% CD34^+^-enriched cell population (human Diamond CD34 isolation kit, Miltenyi Biotec, Bergisch Gladbach, Germany 130-094-531).

For the xenotransplantation experiments in the humanised mice, a single dosage of 2.5 × 10^5^ human PB CD34^+^ cells were injected retro-orbitally with 100 μl cell suspension into 2–3 months old NBSGW mice. Human conjugated antibodies used for assessing kinetics derived from Biolegend (1:100 dilution); huCD45-Brv421 (368522), huCD19-510Br (302241), huCD3-PE (300308), huCD14-FITC (325604), CD34-APC/Cy7 (343514), CD11b-PercP/Cy5.5 (301327), huCD31-PercP/Cy5.5 (303132), huCD73-APC (344005), huCD105-PE (323205) and huPDGFRα-PE/Cy7 (323508).

### Flow cytometry analysis and human HSC and MPP isolation

Freshly isolated human CD34^+^ and peripheral blood mononuclear cells (PBMC) (2 × 10^6^ cells/sample) were directly immunolabelled with PE anti-human CD34 (clone 561, 1:200), FITC anti-human CD45RA (clone HI100, 1:200), APC anti-human CD38 (clone HB-7, 1:200), PE/Cy7 anti-human CD90 (clone 5E10, 1:200), Brilliant violet 421 anti-human/mouse CD49f. (clone GoH3, 1:100) and 7-AAD viability staining solution (1:300), all from Biolegend, in 5% FBS in 1× PBS for 30′ at 4 °C. Cells were incubated with the antibodies and viability staining for 30′ at room temperature before washing twice with 1× PBS. Cells were then resuspended in 5% FBS and passed through a 70 μm filter pore tip ensuring single cell-only passage for flow cytometric analysis. Flow Cytometry data analysis was performed using FlowJo™ V10.

### NGS

Human CD34^+^ cells were lysed and RNA was extracted using Qiagen’s Nucleospin RNA XS kit, according to the manufacturer’s protocol. In total, ten healthy (7 PB and 3 BM) and sixteen SLE (6 PB and 10 BM) human samples were used throughout this study concerning RNA-seq experiments. For minimal batch effects, all mRNA-seq experiments were carried out in the Greek Genome Center (GGC) of Biomedical Research Foundation of the Academy of Athens (BRFAA) by the same user. All mRNA-seq libraries were prepared with the Illumina TruSeq RNA v2 kit using 20–120 ng of total RNA (excluding duplicate values). Libraries were checked with the Agilent bioanalyzer DNA1000 chip, quantitated with the Qubit HS spectrophotometric method and pooled in equimolar amounts for Sequencing. 75 bp single-end reads were generated with the Illumina NextSeq500 sequencer.

### NGS data analysis pipeline

Raw reads in FASTQ format were collected and quality control was performed using FASTQC^49^. Low quality bases (q < 30) and adapters were trimmed from the 3’ end of the reads using Cutadapt 1.16^50^. Alignment was performed using the gapped-read STAR 2.6 mapper^51^ against the human genome (hg38). Downstream analysis including quantification using the gencode.v29 annotation gtf file^51^, PCA plots, differential expression analysis using edgeR and heatmaps was performed using SeqMonk 1.44.0 software^52^. Genes with a false discovery rate < 0.05 (FDR- Benjamini and Hochberg correction^53^) were considered significantly differentially expressed (DEG). Gene Ontology (GO) and pathway enrichment analysis as well as enrichment of transcription factor (TF) binding sites using DEG were performed using Toppgene^54^, Cytoscape and ClueGo^55^ tools. GO terms, pathways and TF-binding sites with a FDR < 0.05 were considered statistically significantly enriched.

### Semi-Quantitative PCR

RNA was quantified (by Nanodrop) and retrotranscribed to cDNA (PrimeScript™ RT-PCR Kit—Takara Bio Cat. # RR014A). Real time PCR was performed with KAPA SYBR® FAST qPCR Master Mix (2X) Kit using the following oligonucleotide primers: NRF1 (forward, 5′-CACAGAAAAGGTGCTCAAAGGA-3′; reverse, 5′-CCTGGGTCCATGAAACCCTC-3′), SIRT7 (forward, 5′-CCTGAGCGCGGCCTG-3′; reverse, 5′-GCCTGTGTAGACGACCAAGT-3′), β-actin (forward, 5′-CTCTTCCAGCCTTCCTTCCT-3′; reverse, 5′-AGCACTGTGTTGGCGTACAG-3′). Samples were normalized to β-actin and expression levels were calculated using the 2ΔΔ^−Ct^ method.

### Statistics

Statistical analysis on flow cytometric data was performed using ANOVA T-test using Mann–Whitney or Bonferroni post-hoc test, where appropriate (*p* < 0.05). RNA-seq data statistical analysis was initially performed using EdgeR with a *p* value (< 0.05) and multiple correction testing, for obtaining DEG lists. Downstream analysis involved False Discovery Analysis (FDR) based on Benjamini and Hochberg^53^. For the xenotranplantation inoculation outcome, the GraphPad Prism v8 software was employed.

### Patients and study approval

Informed consent was obtained from all patients and human controls prior to sample collection (IRB protocol number 10/22-6-2017). Table 1 depicts the demographics and diseases characteristics of SLE patients at the time where PB samples were obtained. In all analyses presented here, all human subjects were female apart from three individuals (two SLE and one healthy), identified on relevant figures and figure legends.

BM aspirates were obtained from SLE and healthy controls. BM SLE and healthy aspirates derived from age-matched individuals (set for practical reasons to no more than 10 difference of age was a 10-year age). Patients met the 1997 American College of Rheumatology revised criteria for the classification of SLE^56^.

## Supplementary Information

Supplementary Figures.

Supplementary Table 1.

Supplementary Table 2.
