# Supplementary material for: Patrolling human SLE haematopoietic progenitors demonstrate enhanced extramedullary colonisation; implications for peripheral tissue injury
Source: Sci Rep. 2021 Aug 3;11:15759. doi: 10.1038/s41598-021-95224-y (PMC8333421; doi:10.1038/s41598-021-95224-y)
Supplement: Supplementary file 1 — Supplementary Figures. [file 41598_2021_95224_MOESM1_ESM.pdf]

A

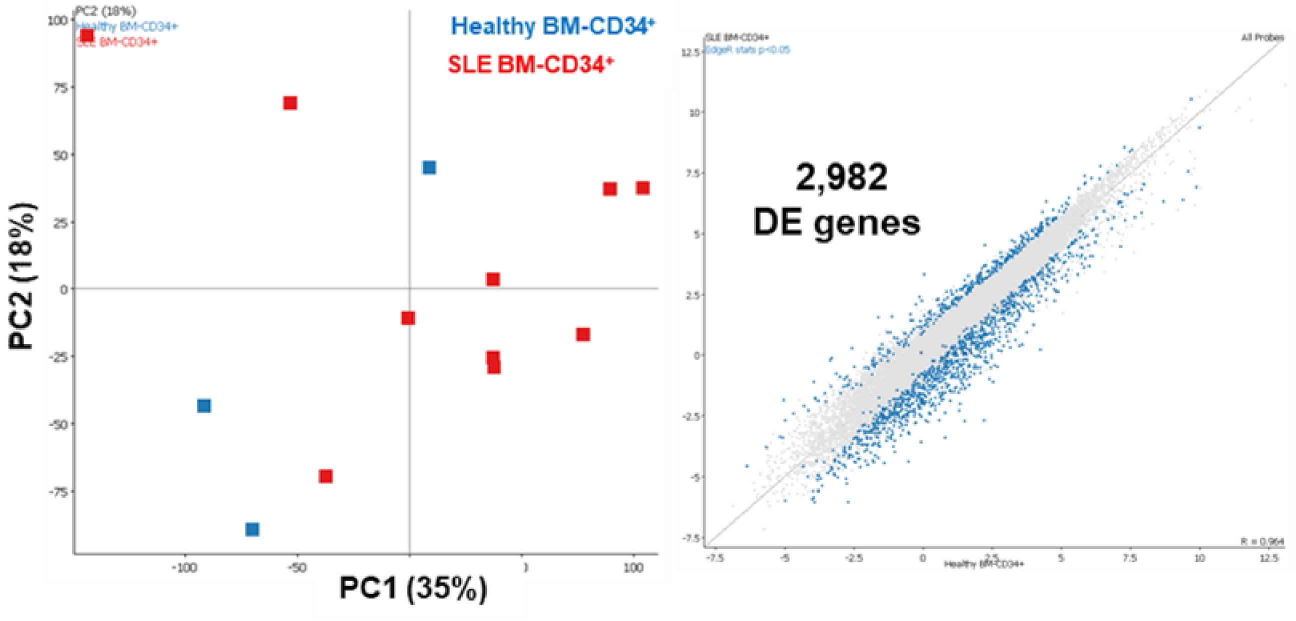

B

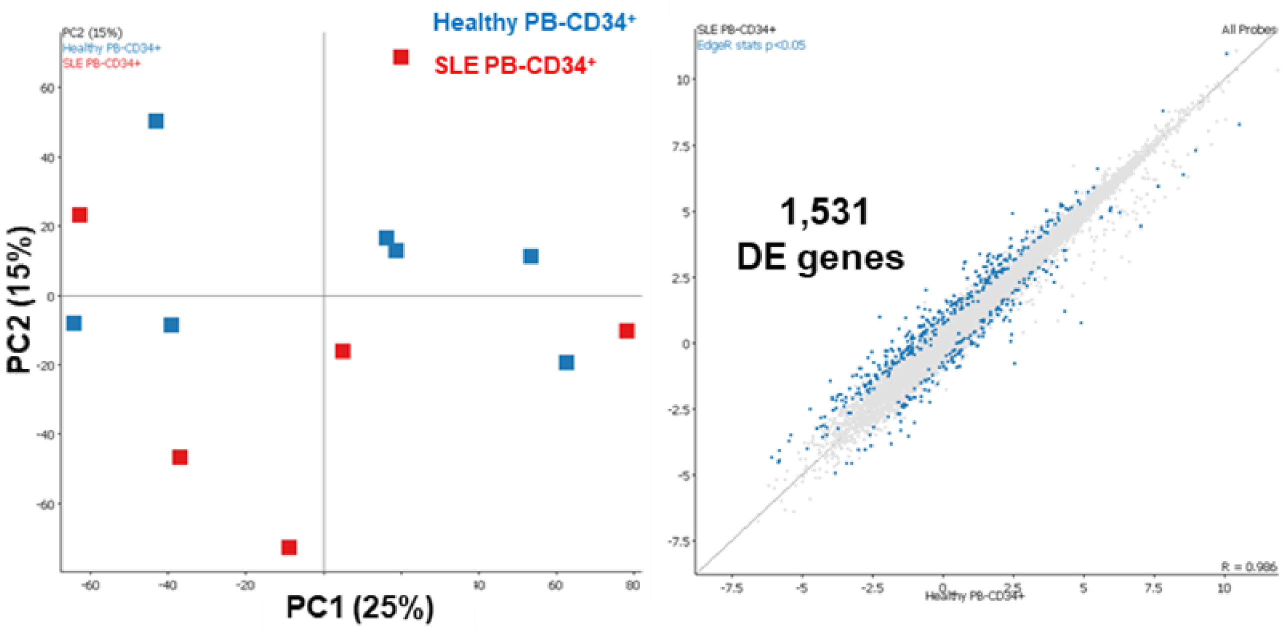

Supplementary Figure 1. PCA analysis and scatter plot of (**A**) Healthy BM vs SLE BM progenitors and (**B**) Healthy PB vs SLE PB progenitors revealed an inability to cluster the two circulating progenitor populations, based on disease status. EdgeR analysis indicated 2,982 DE genes between Healthy and SLE BM-derived CD34$^+$ progenitors, while in PB-derived samples, 1,531 DE genes were detected between Healthy and SLE CD34$^+$ progenitors. Analyses were performed with an FDR<0.05 (Bonferroni and Heidelberg).

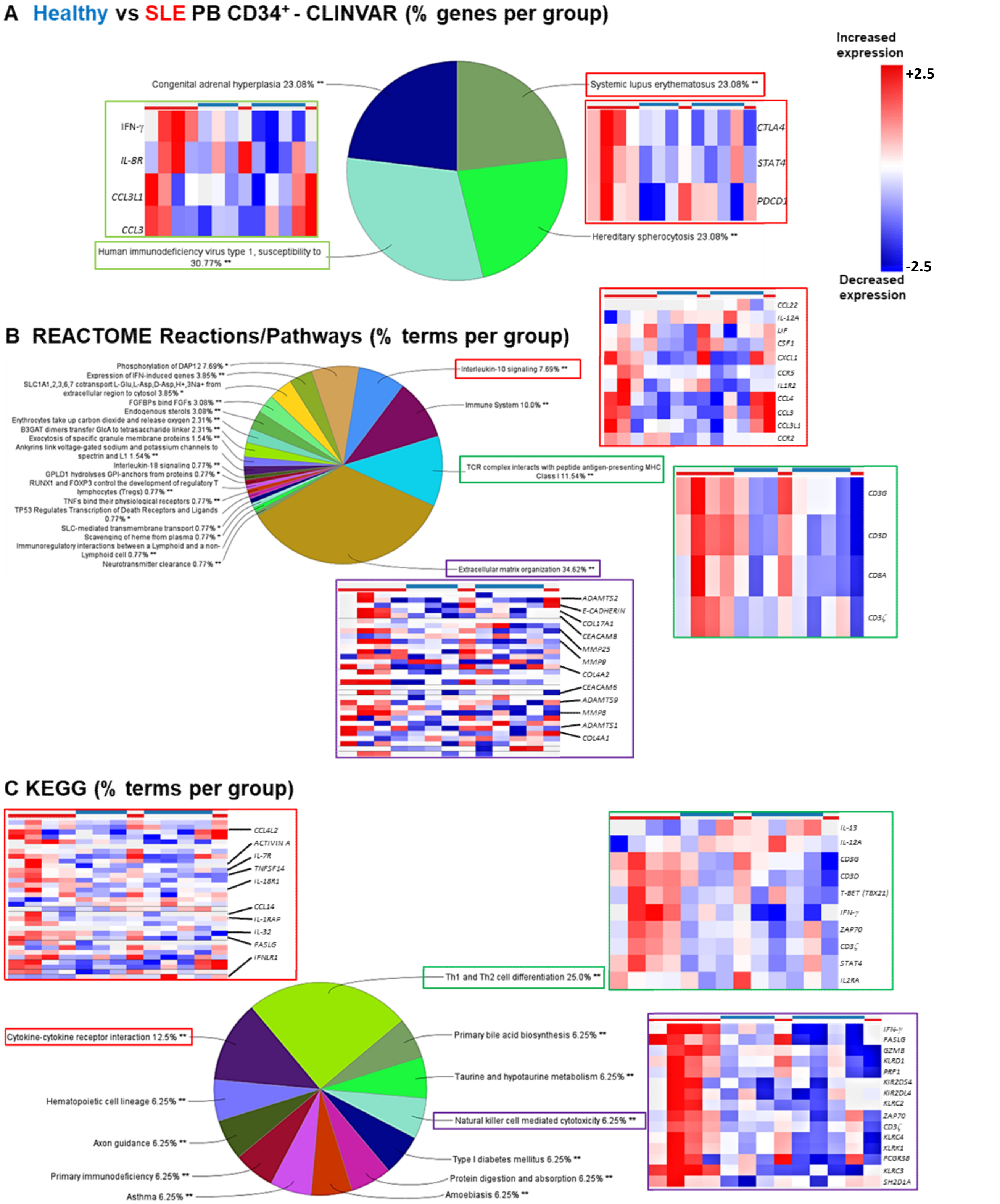

Supplementary Figure 2. SLE PB CD34+ progenitors show upregulated expression of genes involved in extramedullary colonisation. (**A**) CLINVAR analysis of DEG between Healthy PB (blue) vs SLE PB (red) CD34+ progenitors with relevant heatmaps of the bracketed GO terms. (**B**) REACTOME Reactions/Pathways analysis of DEG between Healthy PB (blue) vs SLE PB (red) CD34+ progenitors with relevant heatmaps of the bracketed GO terms. (**C**) KEGG analysis of DEG between Healthy PB (blue) vs SLE PB (red) CD34+ progenitors with relevant heatmaps of the bracketed GO terms. All analyses were performed with an FDR<0.05. *p<0.05, **p<0.01.

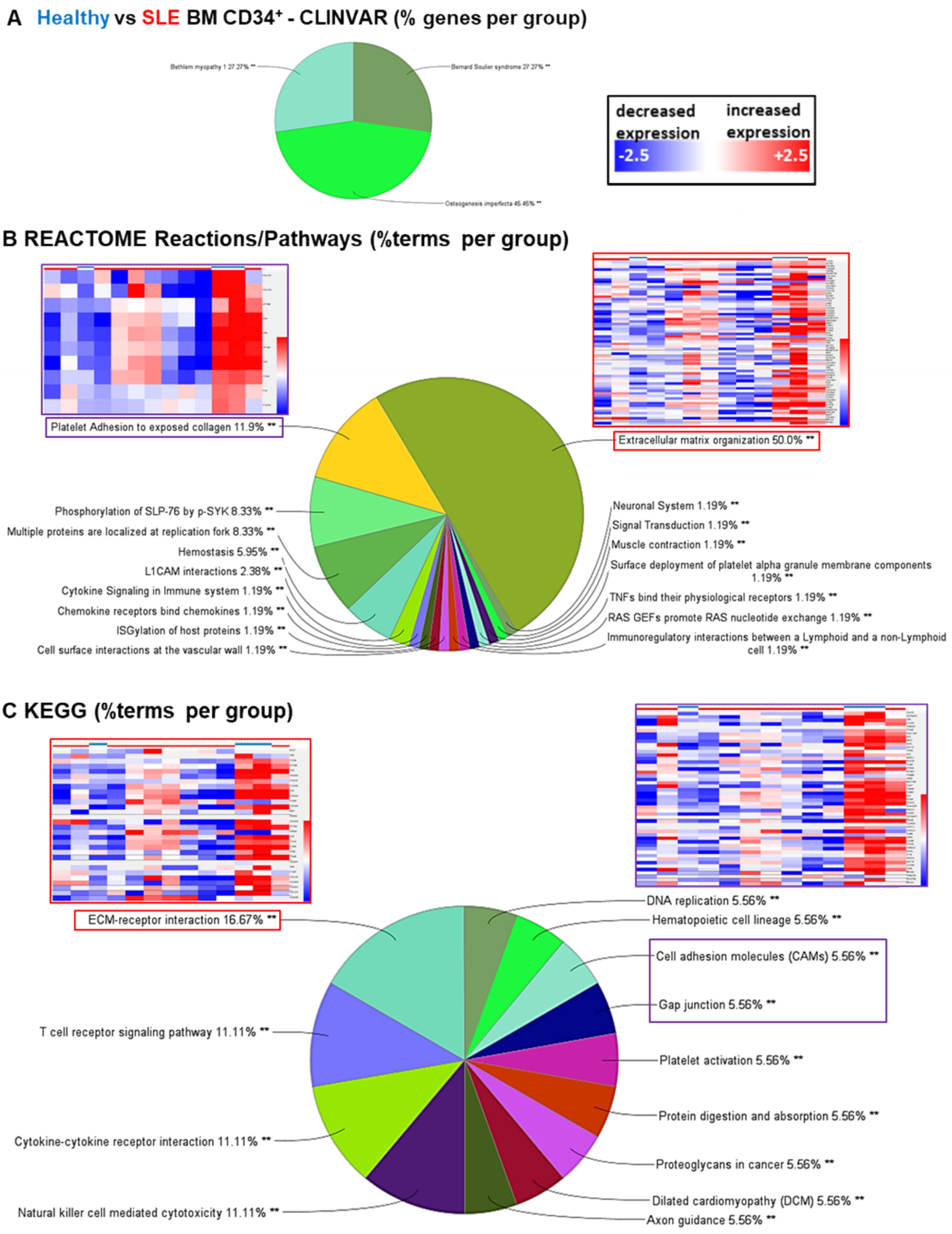

Supplementary Figure 3. (**A**) CLIVAR analysis of DE genes between Healthy BM-derived (Blue) and SLE BM (red) CD34+ progenitors. Analysis were performed with a p<0.05 with an FDR correction. (**B**) REACTOME Reactions/Pathways analysis of DE genes between Healthy BM and SLE BM $CD34^{+}$ progenitors. All analyses were performed with a p<0.05 with an FDR correction. (**C**) KEGG Reactions analysis of DE genes between Healthy BM and SLE BM $CD34^{+}$ progenitors. Analysis were performed with a p<0.05 with an FDR correction. Analyses were performed with an FDR<0.05 (Bonferroni and Heidelberg). *p<0.05, **p<0.01.

A CLINVAR and KEGG (SLE BM vs SLE PB)

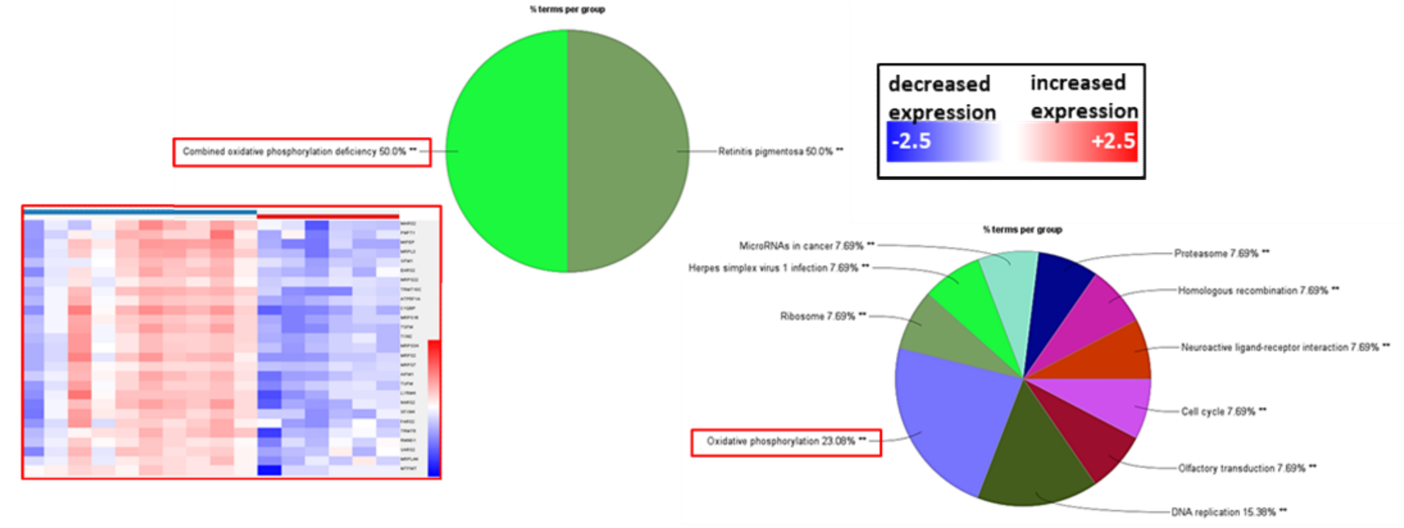

B CLIVAR and KEGG (Healthy BM vs Healthy PB)

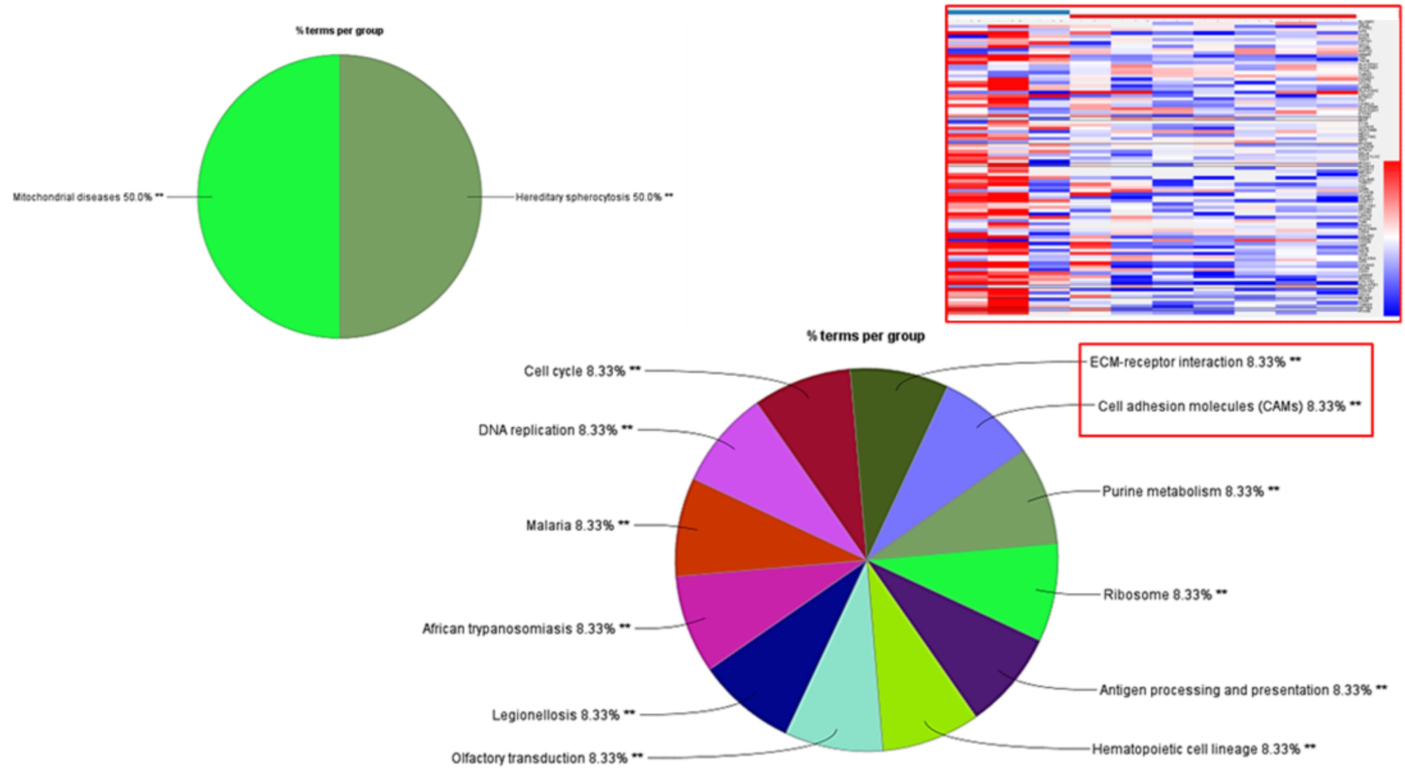

Supplementary Figure 4. (**A**) CLINVAR analysis in SLE PB vs BM and (**B**) Healthy PB vs BM. (C, D) PCA analysis and scatter plot of Healthy BM vs SLE BM progenitors and Healthy PB vs SLE PB progenitors revealed an inability to cluster the two circulating progenitor populations, based on disease status. EdgeR analysis indicated 2,982 DE genes between Healthy and SLE BM-derived CD34$^+$ progenitors, while in PB-derived samples, 1,531 DE genes were detected between Healthy and SLE CD34$^+$ progenitors. Analyses were performed with an FDR<0.05 (Bonferroni and Heidelberg). *p<0.05, **p<0.01.

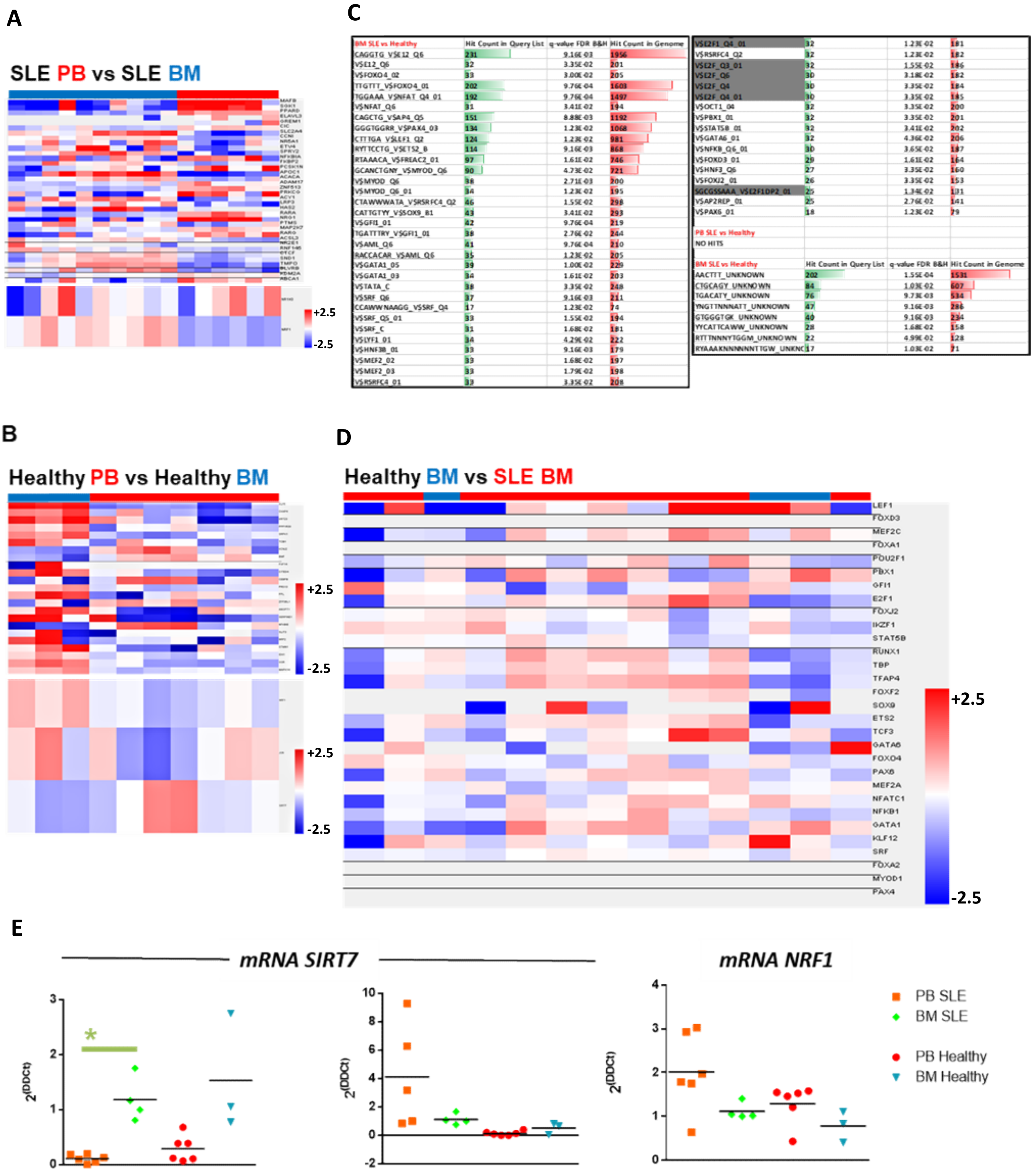

BM SLE vs Healthy	Hit Count in Query List	q-value FDR B&H	Hit Count in Genome
CAGGTG_V$E12_Q6	231	9.16E-03	1956
V$E12_Q6	32	3.35E-02	201
V$FOXO4_02	33	3.00E-02	205
TTGTTT_V$FOXO4_01	202	9.76E-04	1603
TGGAAA_V$NFAT_Q4_01	192	9.76E-04	1497
V$NFAT_Q6	31	3.41E-02	194
CAGCTG_V$AP4_Q5	151	8.88E-03	1192
GGGTGGRR_V$PAX4_03	134	1.23E-02	1068
CTTTGA_V$LEF1_Q2	124	1.23E-02	981
RYTTCCTG_V$ETS2_B	114	9.16E-03	868
RTAAACA_V$FREAC2_01	97	1.61E-02	746
GCANCTGNY_V$MYOD_Q6	90	4.73E-02	721
V$MYOD_Q6	38	2.71E-03	200
V$MYOD_Q6_01	34	1.23E-02	195
CTAWWWATA_V$RSRFC4_Q2	46	1.55E-02	298
CATTGTYY_V$SOX9_B1	43	3.41E-02	293
V$GFI1_01	42	9.76E-04	219
TGATTTRY_V$GFI1_01	38	2.76E-02	244
V$AML_Q6	41	9.76E-04	210
RACCACAR_V$AML_Q6	35	1.23E-02	205
V$GATA1_05	39	1.00E-02	229
V$GATA1_03	34	1.61E-02	203
V$TATA_C	38	3.35E-02	248
V$SRF_Q6	37	9.16E-03	211
CCAWWNAAGG_V$SRF_Q4	17	1.23E-02	74
V$SRF_Q5_01	33	1.55E-02	194
V$SRF_C	31	1.68E-02	181
V$LYF1_01	34	4.29E-02	222
V$HNF3B_01	33	9.16E-03	179
V$MEF2_02	33	1.68E-02	197
V$MEF2_03	33	1.79E-02	198
V$RSRFC4_01	33	3.35E-02	208
V$E2F1_Q4_01	32	1.23E-02	181
V$RSRFC4_Q2	32	1.23E-02	182
V$E2F_Q3_01	32	1.55E-02	186
V$E2F_Q6	30	3.18E-02	182
V$E2F_Q4	30	3.35E-02	184
V$E2F_Q4_01	30	3.35E-02	185
V$OCT1_04	32	3.35E-02	200
V$PBX1_01	32	3.35E-02	201
V$STAT5B_01	32	3.41E-02	202
V$GATA6_01	32	4.36E-02	206
V$NFKB_Q6_01	30	3.65E-02	187
V$FOXD3_01	29	1.61E-02	164
V$HNF3_Q6	27	3.35E-02	160
V$FOXJ2_01	26	3.35E-02	153
SGCGSSAAA_V$E2F1DP2_01	25	1.34E-02	131
V$AP2REP_01	25	2.76E-02	141
V$PAX6_01	18	1.23E-02	79
PB SLE vs Healthy			
NO HITS			
BM SLE vs Healthy	Hit Count in Query List	q-value FDR B&H	Hit Count in Genome
AACTTT_UNKNOWN	202	1.55E-04	1531
CTGCAGY_UNKNOWN	84	1.03E-02	607
TGACATY_UNKNOWN	76	9.73E-03	534
YNGTTNNNATT_UNKNOWN	47	9.16E-03	286
GTGGGTGK_UNKNOWN	40	9.16E-03	234
YYCATTCAWW_UNKNOWN	28	1.68E-02	158
RTTTNNNYTGGM_UNKNOWN	22	4.99E-02	128
RYAAAKNNNNNNTTGW_UNKNC	17	1.03E-02	71

Supplementary Figure 5. (**A**) Heatmaps of NRF1 and NR1H3 expression in SLE PB (red) and SLE BM (healthy), along with the DEGs that are affected by NRF1 expression. (**B**) Heatmaps of NRF1 and NR1H3 expression in Healthy PB (red) and Healthy BM (blue), along with the DE genes that are affected by CEBPB. (**C**) Table indicating the predicted TF binding sites (Toppgene software) based on the DE gene input lists for BM SLE vs Healthy, PB SLE vs Healthy and BM SLE vs Healthy (including putative TF binding sites). (**D**) Heatmaps of expression for TFs of interest in Healthy BM (blue) and SLE BM (red). All analyses were performed with a p<0.05 with an FDR (Bonferroni and Heidelberg) correction. (**E**) Real-time PCR graphs for NRF1 variants (ENST00000311967.6, ENST00000223190.8) and SIRT7 variants (ENST00000572902.5, SIRT7-210 and ENST00000328666.11, SIRT7-201) in all four groups. The expression of NRF1 was not statistically different in any of the four groups. Some SIRT7 variants were downregulated significantly in SLE PB in comparison to SLE BM CD34+ progenitors. One-way ANOVA with Bonferroni's post-hoc test, *p<0.05, n=3-6.

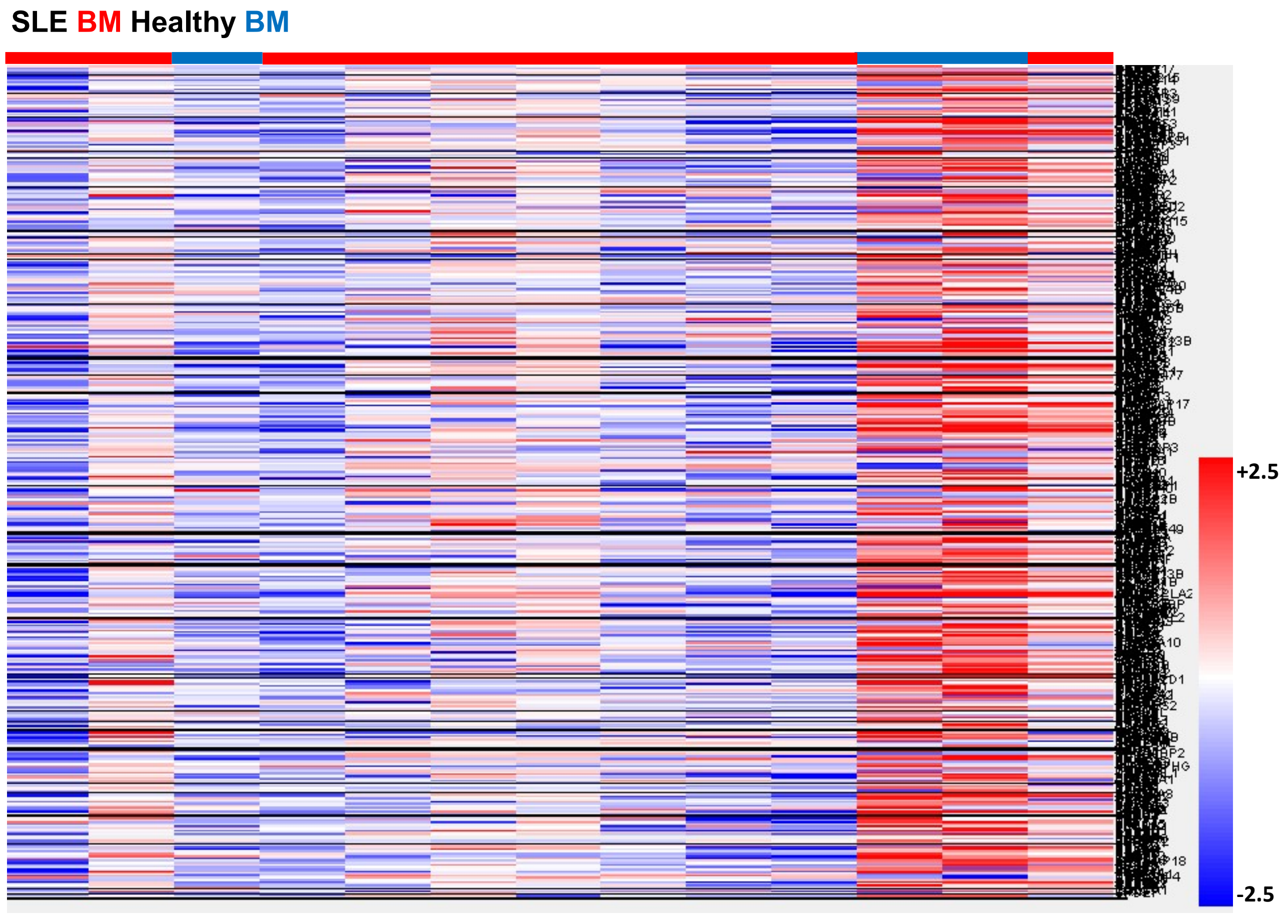

Supplementary Figure 6. Heatmap of predicted TF-binding sites related DEGs between SLE BM (red) and Healthy BM (healthy).

Supplementary Figure 7 **Kokkinopoulos *et al.***

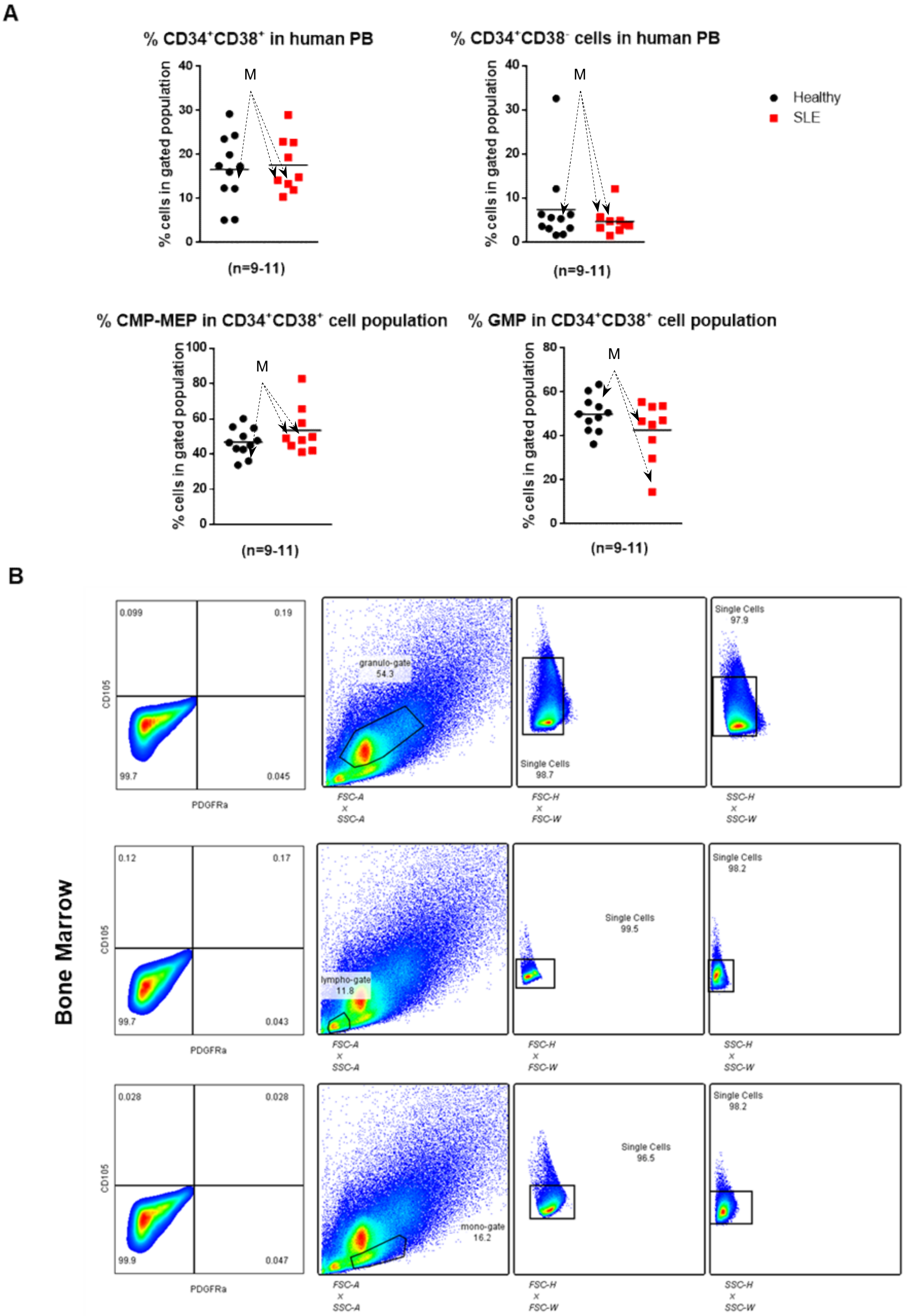

Supplementary Figure 7. (**A**) Peripheral blood CMP-MEP and GMP numbers remain the same in PB Healthy vs SLE. X-Y graphs showing the percentage of $CD34^{+}CD38^{+}$, $CD34^{+}CD38^{-}$, CMP-MEP and GMP in PBMC-gated and $CD34^{+}CD38^{-}$-gated cell populations. Healthy n=9, SLE n=11, Student's T-test. M=male subjects. (**B**) Representative diagrams of the gating strategy employed for examining human MPP-derived cell populations in the bone marrow of humanised mice. A similar strategy was used upon examination of kidney and spleen tissue.

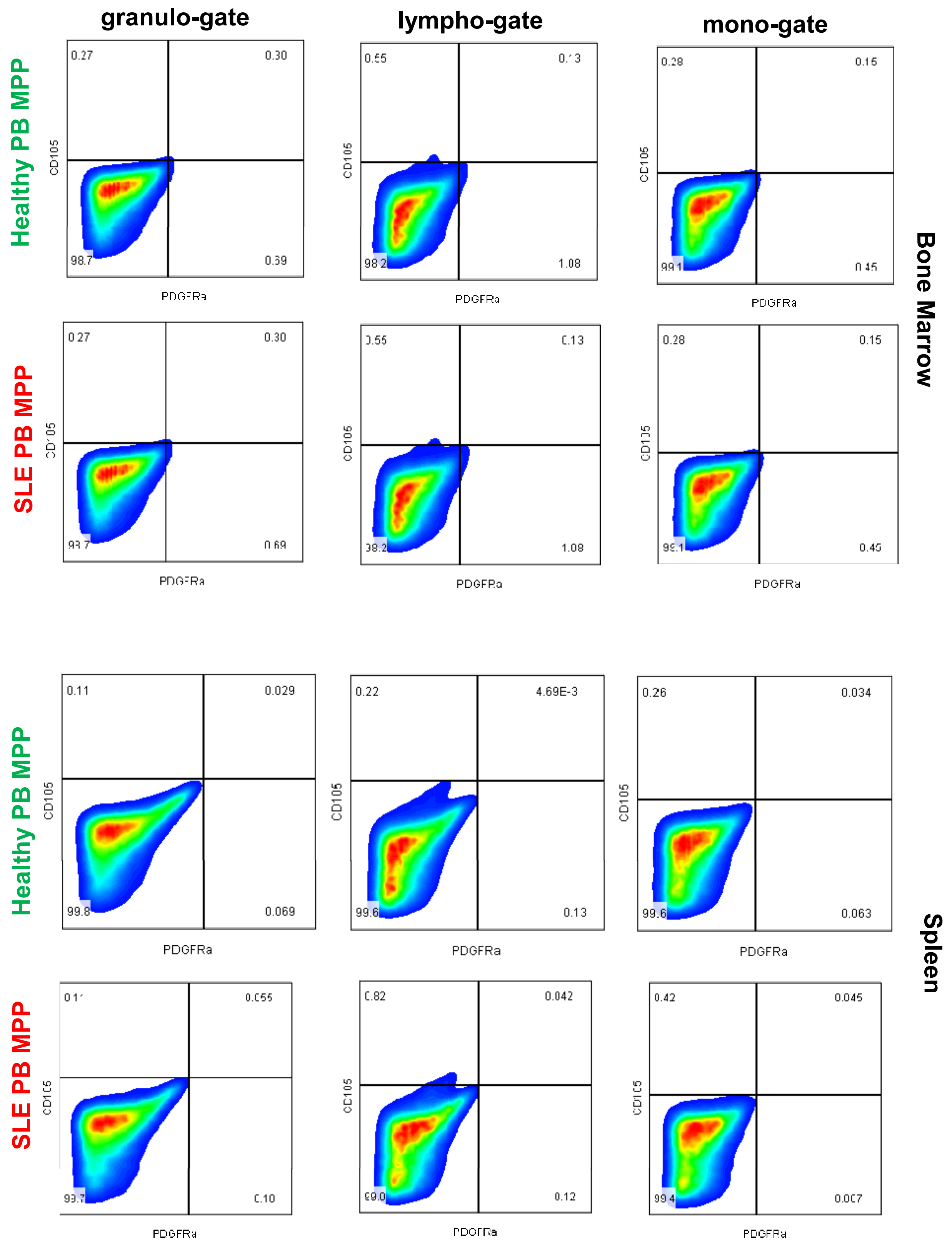

Supplementary Figure 8. MPPs were not shown to colonise the bone marrow or the spleen. Mice were sacrificed at 5 weeks and bone marrow and spleen cells were subjected into immunostaining with antibodies against human-only surface markers for CD105 and PDGFRα, in order to assess human cell presence. Lympho-gates, granulo-gates and mono-gates represent different immune cell subpopulations and therefore examined separately, according to their FSC/SSC readout. N=4.
